# Evaluating clinical periodontal measures as surrogates for bacterial exposure: The Oral Infections and Vascular Disease Epidemiology Study (INVEST)

**DOI:** 10.1186/1471-2288-10-2

**Published:** 2010-01-07

**Authors:** Ryan T Demmer, Panos N Papapanou, David R Jacobs, Moïse Desvarieux

**Affiliations:** 1 Department of Epidemiology, Mailman School of Public Health, Columbia University, New York, NY, USA; 2 Division of Periodontics, Section of Oral and Diagnostic Sciences, College of Dental Medicine, Columbia University, New York, NY, USA; 3 Division of Epidemiology and Community Health, School of Public Health, University of Minnesota, Minneapolis, MN, USA; 4 Department of Nutrition, University of Oslo, Oslo, Norway; 5 INSERM, UMR-S 707, Paris, F-75012; Universite Pierre et Marie Curie-Paris6, UMR S 707, Paris, F-75012, France; 6 Ecole des hautes etudes en sante publique, Paris et Rennes, France

## Abstract

**Background:**

Epidemiologic studies of periodontal infection as a risk factor for cardiovascular disease often use clinical periodontal measures as a surrogate for the underlying bacterial exposure of interest. There are currently no methodological studies evaluating which clinical periodontal measures best reflect the levels of subgingival bacterial colonization in population-based settings. We investigated the characteristics of clinical periodontal definitions that were most representative of exposure to bacterial species that are believed to be either markers, or themselves etiologic, of periodontal disease.

**Methods:**

706 men and women aged ≥ 55 years, residing in northern Manhattan were enrolled. Using DNA-DNA checkerboard hybridization in subgingival biofilms, standardized values for *Aggregatibacter actinomycetemcomitans, Porphyromonas gingivalis, Treponema denticola *and *Tannerella forsythia *were averaged within mouth and summed to define "bacterial burden". Correlations of bacterial burden with clinical periodontal constructs defined by the severity and extent of attachment loss (AL), pocket depth (PD) and bleeding on probing (BOP) were assessed.

**Results:**

Clinical periodontal constructs demonstrating the highest correlations with bacterial burden were: i) percent of sites with BOP (r = 0.62); ii) percent of sites with PD ≥ 3 mm (r = 0.61); and iii) number of sites with BOP (r = 0.59). Increasing PD or AL severity thresholds consistently attenuated correlations, i.e., the correlation of bacterial burden with the percent of sites with PD ≥ 8 mm was only r = 0.16.

**Conclusions:**

Clinical exposure definitions of periodontal disease should incorporate relatively shallow pockets to best reflect whole mouth exposure to bacterial burden.

## Background

There are several models for studying infectious origins of cardiovascular disease(CVD) [1]. Many models rely on serum antibody titer to define infectious exposure. This has the advantage of capturing historical infectious exposure, but the possible disadvantage of not requiring the presence of active infection at the time of measurement. Another method of assessing infectious bacterial exposure is to directly measure bacterial colonization levels. In this regard, one infection model that may be useful is periodontal disease, because active bacterial infections with inflammatory consequences are often present for years and are easily accessible in the subgingiva[2-5].

However, microbiological assessment is resource intensive and is rarely done in epidemiologic studies investigating periodontal-CVD associations. Consequently, studies exploring associations between periodontal infection and CVD frequently use one of a wide variety of whole mouth summaries of clinical periodontal measures (i.e. pocket depth, attachment loss and bleeding on probing) as a surrogate measure of underlying microbiology. Methodological research is needed to clarify which clinical periodontal constructs act as surrogates for such bacterial infection. In doing so, hypothesis testing in future studies can be more specifically focused on infectious exposure as opposed to clinical periodontal characteristics which can be the result of non-infectious exposures such as smoking[6-8], diabetes[9], or endodontic lesions[10].

In this paper we investigated how well whole mouth clinical periodontal constructs reflect exposure to bacteria that are associated with risk of periodontal disease.

## Methods

The Oral Infections and Vascular Disease Epidemiology Study (INVEST) is a randomly sampled prospective population-based cohort study investigating whether oral infections increase the risk of carotid atherosclerosis and stroke[11]. Briefly, 1056 subjects were randomly selected from Northern Manhattan, an area between 145^th ^Street and 218^th ^Street, bordered westward by the Hudson River, and separated eastward from the Bronx by the Harlem River. Hispanics, Blacks, and Whites live together in this area and have similar access to medical care. The selection process was derived from the Northern Manhattan Study (NOMAS) in which patients are also enrolled[12].

Of 841 dentate participants enrolled at baseline, both clinical periodontal exams and subgingival plaque samples were available for 706 subjects, who entered these analyses.

The Institutional Review Board at Columbia University approved the study, and all subjects provided informed consent.

### Dental history and oral examination

Subjects were interviewed and examined by trained research assistants and calibrated dental examiners[4,11]. Teeth were counted and localized. Plaque samples in the two most posterior teeth (mesiolingual in the upper jaw and mesiobuccal in the lower jaw) were collected before probing. Assessment of periodontal status for all teeth present included presence/absence of dental plaque, probing depth in millimeters, and location of the gingival margin in relation to the cementoenamel junction at six locations for each tooth (mesiobuccal, midbuccal, distobuccal, mesiolingual, midlingual and distolingual) using a UNC-15 manual probe with ruler markings at each millimeter from 1 to 12 (HuFriedy, Chicago, IL). Attachment loss was calculated as the sum of pocket depth and gingival margin.

### Subgingival plaque collection and laboratory processing

A maximum of eight subgingival plaque samples (mean 7; median 8) were collected from each subject by a single scaling stroke from the base of the periodontal pocket using a sterile Gracey curette. Samples were collected from the two most posterior teeth in each quadrant (mesiopalatal sites in the maxilla and mesiobuccal sites in the mandible). The collected plaque mass from each site was transferred into an individual Eppendorf tube containing 200 μl of sterile T-E buffer (10 mM Tris HCl, 1.0 mm EDTA, pH 7.6). The tubes were immediately transferred into the laboratory and the plaque pellet was re-suspended, vigorously vortexed, and 200 μl of a 0.5 M NaOH solution were added. The samples were kept at +4°C until immobilization onto nylon membranes, within a few days from sample collection.

A total of 4,922 bacterial plaque samples (collected from n = 706 participants) are included presently.

Four bacterial species (*Aggregatibacter actinomycetemcomitans*, *Porphyromonas gingivalis*, *Tannerella forsythia *and *Treponema denticola*) were assessed from each subgingival plaque sample using checkerboard DNA-DNA hybridization as previously described[4,13]. These 4 species were selected because there is strong evidence that they are either markers of, or themselves causal agents for, periodontal disease [4,13].

### Statistical analysis

All analyses were performed in PC-SAS for Windows 9.1 and R for Windows 2.2.1.

Creation of Bacterial Burden: Laboratory analysis provides a relative quantity of individual bacterial species for each subgingival plaque sample by comparison to known standards; these distributions are strongly skewed towards larger values and it is not known whether absolute amounts of different species are comparable. Bacterial values were therefore averaged within mouth, natural log transformed and standardized by dividing each respective natural log transformed bacterial value by the population standard deviation for the respective species. One standard deviation on the log scale was treated as equivalent across bacteria. For each person, exposure to periodontal bacteria was quantified by summing the standardized values for the four bacteria *A. actinomycetemcomitans, P. gingivalis, T. forsythia *and *T. denticola *to create a bacterial burden (BB) score[4]. Bacterial burden scores are presented in standard deviation units (SDU).

#### Creation of Whole Mouth Clinical Periodontal Constructs

We created a series of summary clinical periodontal constructs[14] reflecting both the extent and severity of current periodontal disease (as measured by PD and BOP) and the past cumulative experience to periodontitis (as measured by AL). The terms "extent" and "severity" were adapted from the method reported by Carlos et al. in defining the extent and severity index (ESI)[15].

#### Constructs based on a combination of extent and severity

We considered severity thresholds of 2 - 8 mm for PD or AL. For each severity threshold, disease extent was defined by various metrics yielding multiple constructs based on a combination of extent and severity of clinical periodontal disease as follows: 1) number of sites with PD or AL ≥ specified severity thresholds; 2) percent of sites with PD or AL ≥ specified severity thresholds; 3) sum of PD or AL beyond the specified severity thresholds; 4) mean PD or AL beyond specified severity thresholds; 5) number of sites with PD or AL equal to specified severity thresholds; 6) percent of sites with PD or AL equal to specified severity thresholds. We also created the aforementioned constructs using only periodontal measurements taken from interproximal sites.

#### Definitions based on extent only

The overall mean PD and AL allowed clinical measures from all sites to directly contribute information regarding periodontal disease. Likewise, for BOP we calculated the number and percent of measured sites that bled on probing.

#### Post hoc Definitions

Based on the findings from the aforementioned definitions, we created a post hoc periodontal definition based on a site by site combination of bleeding on probing status and whether or not the pocket was ≥ 3 mm as follows: i) sites with PD ≤ 3 mm received a value = 0 (regardless of bleeding status); ii) sites with PD ≥ 3 and no BOP received a value = 1; iii) sites with PD ≥ 3 and BOP received a value = 2. Finally, we took the whole mouth average of these values to represent the individual. The purpose of this definition was to see if the combination of information from two variables could enhance observed associations between clinical periodontal definitions and bacterial burden.

Correlations were run between all candidate periodontal variables and the dependent variable "bacterial burden". We tested statistical significance of differences in correlations among the periodontal constructs using the "compOverlapCorr" package in R (developed by Li and Zhu, 2006; see R user's manual). This is based on a method for comparing correlated correlations between variables (Y,X1) and (Y,X2), described by Meng et al[16]. The correlations are correlated because of a shared dependent variable, Y, as well as a correlation between X1 and X2. The algebraic formula for the Z test comparing the difference between two sample correlation coefficients (i.e., r_Y,X1 _and r_Y,X2_), follows: (z_r1_-z_r2_)*square root of ((N-3)/(2(1-r_x_)h)), where N = sample size; z_ri _is the Fisher z-transformed r_Y,Xi_; r_x _is the correlation between the two predictor variables X1 and X2; r^2^_1,2 _mean = (r^2^_Y,X1_+r^2^_Y,X2_)/2, h = 1-f(r^2^_1,2 _mean)/(1- r^2^_1,2 _mean); f = (1-r_x_)/(2(1- r^2^_1,2 _mean)) which was required to be ≤ 1. This formulation shows that statistical significance of differences between two correlation coefficients r_Y,X1 _and r_Y,X2 _depends strongly on the correlation between the two periodontal constructs being compared r_X1,X2_.

All analyses were conducted among subgroups according to either smoking status, diabetes status or gender to inform the potential for effect modification or confounding. This analysis is methodological as opposed to etiological in nature. Therefore, we present p-values for the reader's information but encourage the reader to consider the overall trends as opposed to any specific tests of statistical significance as these p-values are not adjusted for multiple comparisons and the data are observational.

## Results

INVEST participants are tri-ethnic (57% Hispanic, 23% Black and 20% White) with an average age ± SD of 69 ± 9 years. Women comprise 60% of this sample and on average participants have lost 14 ± 8 teeth (including 3^rd ^molars).

Bacterial burden was normally distributed with mean ± SD of 32 ± 4 SDU. Summary clinical periodontal definitions became highly skewed with increasing severity thresholds because few participants exhibited high AL or deep PD (Table 1). For example, 99% of participants had at least one periodontal site with a PD ≥ 3 mm while only 11% had at least one site with PD ≥ 8 mm. Among the 89% of participants with no PD sites ≥ 8 mm substantial variation in periodontal status remained; the number of sites with PD ≥ 3 mm ranged from 0-150 (median = 32) and the percent of sites with PD ≥ 3 mm ranged from 0%-97% (median = 36%).

**Table 1:** Distributions for *Selected Clinical Periodontal Constructs (n = 706).

Variable	25^th^	Median	Mean	75^th^	Std Dev
**%AL ≥ 3**	28	57	56	86	32
**%AL ≥ 6**	0	3	12	14	19
**%AL ≥ 8**	0	0	3	2	10
					
**%PD ≥ 3**	19	40	43	67	27
**%PD ≥ 6**	0	0	2	0	6
**%PD ≥ 8**	0	0	0.5	0	2
					
**%BOP**	3	13	33	50	46
**#BOP**	2	11	27	35	36

Abbreviations: AL: Attachment loss PD: Pockect depth BOP: bleeding on probing 25^th ^and 75^th ^denote percentiles. % refers to percent of sites per mouth. # refers to the number of sites per mouth. *Note, distributional patterns across increasing severity cut points were similar for all clinical periodontal constructs (data not shown).

Clinical periodontal definitions showed a wide range of correlations with one another. Definitions utilizing similar extent or severity criteria were highly correlated. For example, definitions summarizing the percent of sites with PD ≥ 4 or 5 mm had a correlation of r = 0.90, while the percent of sites with PD ≥ 2 or 8 mm were only correlated at r = 0.17. Tables 2 and 3 present correlation matrices for selected periodontal definitions.

**Table 2:** Correlations between Clinical Periodontal Constructs (n = 706)

	%PD ≥ 2	%PD ≥ 3	%PD ≥ 4	%PD ≥ 5	%PD ≥ 6	%PD ≥ 7	%PD ≥ 8
**%PD ≥ 2**	1	0.78	0.51	0.39	0.26	0.19	0.17
**%PD ≥ 3**		1	0.76	0.62	0.43	0.33	0.29
**%PD ≥ 4**			1	0.88	0.69	0.58	0.51
**%PD ≥ 5**				1	0.86	0.75	0.67
**%PD ≥ 6**					1	0.92	0.81
**%PD ≥ 7**						1	0.84
**%PD ≥ 8**							1

Correlations between selected clinical periodontal constructs holding extent definition (percent of sites with pocket depth) constant and allowing the severity threshold to vary.

**Table 3:** Correlations between Clinical Periodontal Constructs (n = 706)

	#PD ≥ 3	%PD ≥ 3	Mean PD ≥ 3	Sum PD ≥ 3	#AL ≥ 3	%AL ≥ 3	MeanAL ≥ 3	Sum AL ≥ 3
**#PD ≥ 3**	1	0.68	0.34	0.90	0.80	0.34	0.04	0.67
**%PD ≥ 3**		1	0.53	0.70	0.51	0.72	0.48	0.60
**Mean PD ≥ 3**			1	0.63	0.23	0.32	0.56	0.48
**Sum PD ≥ 3**				1	0.71	0.38	0.25	0.80
**#AL ≥ 3**					1	0.55	0.09	0.84
**%AL ≥ 3**						1	0.58	0.64
**Mean AL ≥ 3**							1	0.49
**Sum AL ≥ 3**								1

Correlations between selected clinical periodontal constructs, holding severity threshold constant and allowing the extent definition to vary.

# refers to number of sites/mouth beyond 3 mm severity threshold.

% refers to percent of sites/mouth beyond 3 mm severity threshold.

Mean refers to mean pocket depth among sites ≥ 3 mm severity threshold.

Sum refers to cumulative pocket depth among sites ≥ 3 mm severity threshold.

Correlations between bacterial burden and several clinical periodontal definitions are shown in Table 4. The strongest correlations were observed for percent of sites bleeding on probing (r = 0.62), percent of sites with PD ≥ 3 mm (r = 0.61) and number of sites bleeding on probing (r = 0.59). Pair-wise comparisons showed no statistically significant differences between any of these three correlation coefficients (all p > 0.10). Comparisons between correlations for any of these three definitions and any other whole-mouth definition in this analysis yielded a p-value of < 0.05. The highest correlation involving AL was 0.48. The post hoc definition representing a combination of PD ≥ 3 and presence of BOP yielded a marginally stronger correlation with bacterial burden (r = 0.64).

**Table 4:** Correlations between Bacterial Burden and Selected Clinical Periodontal Constructs (n = 706)

	Attachment Loss Extent Constructs				Pocket Depth Extent Constructs			
**Severity Threshold**	***#AL ≥ Threshold***	**%AL ≥ Threshold**	***Sum AL ≥ Threshold***	**Mean AL ≥ Threshold**	***#PD ≥ Threshold***	**%PD ≥ Threshold**	***Sum PD ≥ Threshold***	**Mean PD ≥ Threshold**
**2 mm**	*0.39 (A,C)*	0.48 (A)	*0.47 (A)*	0.30 (A)	*0.16 (A,E,F)*	0.42 (A)	*0.40 (A,C)*	0.49 (A)
**3 mm**	*0.48 (B)*	0.48 (A)	*0.46 (A)*	0.21 (B)	*0.51 (B)*	0.61 (B)	*0.48 (B)*	0.25 (B,D)
**4 mm**	*0.40 (A)*	0.30 (B)	*0.38 (B)*	0.27 (A)	*0.39 (C)*	0.39 (A)	*0.39 (A)*	0.34 (C)
**5 mm**	*0.39 (A)*	0.29 (B)	*0.34 (C)*	0.23 (B)	*0.39 (C)*	0.38 (A)	*0.35 (C)*	0.28 (D)
**6 mm**	*0.33 (C)*	0.26 (B)	*0.29 (D)*	0.25 (B)	*0.28 (D)*	0.26 (C)	*0.24 (D)*	0.27 (D)
**7 mm**	*0.29 (D)*	0.23 (C)	*0.25 (E)*	0.26 (A,B)	*0.18 (E)*	0.20 (D)	*0.19 (E)*	0.18 (E)
**8 mm**	*0.28 (D)*	0.24 (B,C)	*0.23 (F)*	0.24 (A,B)	*0.14 (F)*	0.16 (D)	*0.15 (F)*	0.15 (E)

Bacterial burden is the dependent variable for all 56 correlations presented. All 56 correlation coefficients are statistically significantly different than zero (p-value < 0.0001 for null hypothesis Ho: ρ = 0). Pairwise comparisons were performed between all seven correlation coefficients within each extent periodontal construct (represented in columns), to see if severity threshold impacted strength of correlation (Ho: ρ_1i _= ρ_2i_,); coefficients that do not share a common letter are statistically significantly different (p < 0.05) - based on methods for comparing correlated correlation coefficients from Meng et al.(Meng et al. 1992) For example, the correlation between bacterial burden (the common dependent variable) and %PD ≥ 3 mm (r = 0.61) is statistically significantly different than the correlation between bacterial burden and %PD ≥ 4 mm (r = 0.39).

Results were not meaningfully different among subgroups according to either gender, smoking status or diabetes status.

When using periodontal constructs based on only interproximal periodontal measurements, the results were very similar to those shown in Table 4. For example, the correlation between bacterial burden and the percent of sites with interproximal PD ≥ 3 mm was 0.62 while the correlation for the percent of sites with interproximal PD ≥ 8 mm was 0.15.

Among the definitions that incorporated both severity and extent of clinical periodontal disease, the strongest correlations were observed when severity thresholds in the 2 - 4 mm range were considered (Table 4). It may be appropriate, however, to omit the 2 mm sites, themselves. When considering correlations between bacteria burden and the number or percent of sites *equal *to selected severity criteria, there was a clear demarcation between 2 and 3 mm probing depth or attachment loss. All definitions utilizing a 2 mm criterion were inversely related to bacterial burden, for example having a high percent of sites with PD = 2 mm was suggestive of low bacterial burden (r = -0.55), whereas having a high percent of sites with PD = 3 mm was suggestive of high bacterial burden (r = 0.54).

Both mean AL (r = 0.41) and mean PD (r = 0.52) were also strongly correlated with bacteria burden.

## Discussion

These data demonstrate strong positive associations between several whole mouth clinical periodontal constructs and periodontal bacterial burden. Bacterial burden represents the combined colonization level of four periodontal microbes (*A. actinomycetemcomitans, P. gingivalis, T. denticola *and *T. forsythia*) believed to either cause periodontal disease directly, or to correlate strongly with as yet unidentified causative bacteria [17]. The highest correlations with bacterial burden were consistently observed among the summary clinical constructs utilizing low severity thresholds such as the percent of sites with PD ≥ 3 mm. Bleeding on probing also demonstrated strong positive correlations with bacterial burden.

The observation that clinical constructs utilizing low severity thresholds demonstrated the strongest correlations with bacterial bacteria in this analysis might, at first, appear counterintuitive, since low severity sites are often not regarded as clinical disease. However, these results are not surprising when considering the distributional properties of the clinical constructs utilized presently. We observed that constructs incorporating relatively high severity thresholds demonstrated skewed distributions due to the low prevalence of deep pockets in our cross-sectional epidemiological setting. This low prevalence is potentially due to population characteristics related to access to health care and health behaviors[4]. It is also an expected consequence of severely diseased periodontal sites being predisposed to treatment or removal via tooth loss or extraction. Therefore, in population-based settings such as INVEST, substantial exposure to pathological periodontal microbiology likely occurs in shallow periodontal pockets that do not yet exhibit commonly accepted clinical signs of frank periodontal disease.

This supposition is corroborated by data from other research reports. We have previously found in INVEST, that 77% of ~5,000 examined periodontal sites had a pocket depth of ≤ 3 mm while only 11% of sites were ≥ 5 mm[18]. When considering the ~1200 periodontal sites with high bacteria burden levels (4^th ^quartile), 60% of these sites had pocket depths of ≤ 3 mm[18]. Likewise, data from a Chinese population demonstrated that only 7.5% of 1,864 periodontal sites examined had a probing depth of ≥ 5 mm despite frequent colonization in periodontal sites by species include in our current report[19]. The prevalence of colonization by *A. actinomycetemcomitans, P. gingivalis, T. denticola *and *T. forsythia*, were 38%, 87%, 74% & 73% respectively[19]. Similar results have been published from other populations[20,21].

Results of this nature highlight the potential importance of subclinical periodontal infection in the epidemiological context of periodontal infection and cardiovascular disease risk. Relatively shallow periodontal sites not only have the potential to develop gingivitis and subsequent periodontitis but might also be undergoing subclinical pathological processes that could have systemic effects. Therefore some shallow periodontal sites might actually be considered as nascent disease. Accordingly, in INVEST we have reported that the risk of bleeding on probing associated with high levels of bacterial burden was more pronounced in shallow than in deep periodontal pockets[18]. Moreover, recent research has also shown that low threshold definitions of clinical periodontal disease tend to optimize associations with cardiovascular disease biomarkers[14,22] and two recent publications have reported higher levels of selected periodontal bacteria (including those species studied presently) to be associated with either increased carotid artery intima-media thickness (c-IMT)[4] or increased prevalence coronary heart disease[2].

The finding that pocket depth and bleeding on probing definitions performed as well as, and often better than, attachment loss definitions might have been anticipated when considering that pocket depth and bleeding tend to be better markers of current infection while attachment loss better reflects historical disease. Moreover, attachment loss in buccal sites can be the result of trauma and might have diminish associations with bacterial species, although our results were consistent when using only interproximal sites to create our mouth level clinical definitions.

The definition of a "shallow" periodontal pocket is ambiguous. While the 3 mm severity threshold emerged as an apparent transition from decreased to increased exposure to bacterial burden in our study population, the specific transition threshold might vary across populations where treatment standards and access to dental care differ or behavioral risk factor distributions (i.e., smoking, diabetes or oral hygiene habits) differ. Nevertheless, the overall trends reported presently are likely robust to changing population dynamics. If the current findings are in fact supported more generally in other populations, these concepts might explain some of the variability in reported measures of association between periodontal infection and systemic diseases.

Collecting plaque samples in only 8 sites per mouth might not have adequately represented the microbial profile of the whole mouth. We minimized this potential bias by always collecting plaque samples from the eight most posterior teeth, rather than from the most clinically diseased teeth. Further, although legitimate debate remains in regard to whether *A. actinomycetemcomitans, P. gingivalis, T. denticola *and/or *T. forsythia *are causal of periodontal disease, our decision to use these species as a surrogate for pathological biofilms was based, *a priori*, on the 1996 Consensus Report on Periodontal Diseases: Pathogenesis and Microbial Factors[17] which deemed there to be "Strong Evidence for Etiology" in regard to *A. actinomycetemcomitans*, *P. gingivalis*, and *T. forsythia *(formerly, *B. forsythus*). Our addition of *Treponema denticola *to this construct was also *a priori *and based on the data presented by Socransky *et al*. in 1998 describing the "Red Complex" in which *T. denticola *covaried strongly with *T. Forthysia *and *P. gingivalis*[21]. Subsequently, a substantial body of evidence has emerged - including data from INVEST([18]) - in support of the concept that, at minimum, these microbes are strong correlates of currently unidentified causal microbiology (for original research see [9,20,21,23-28]; for comprehensive reviews see [29-32]). Therefore, taken together, we believe these species are reasonable epidemiologic markers of pathological biofilms, even if these species themselves are at some point determined to be noncausal of periodontal disease. Nevertheless, any conclusions regarding optimal clinical constructs for use as surrogates of periodontal bacteria are limited to the species collected presently. Another limitation to the generalizability of our study is that very few of our participants have minimal or no periodontal disease (gingivitis or periodontitis).

## Conclusions

These findings have implications for studies that posit periodontal disease as a model for infection induced CVD, but that have not collected data on oral bacterial profiles. While the relative risk for bacterial colonization in deep vs. shallow periodontal pockets is high, the prevalence of deep pockets is often low in epidemiological settings. Therefore, in absolute terms, much of the attributable risk from exposure to pathogenic bacteria would likely occur in relatively shallow periodontal pockets. Specifically, our findings highlight the potential importance of using clinical definitions that include less severe periodontal disease when such disease is viewed as a model of infection in studies of systemic disease risk.

## Competing interests

The authors declare that they have no competing interests.

## Authors' contributions

RTD participated in study coordination, statistical analysis and drafted the manuscript. PNP participated in study design and coordination and helped to draft the manuscript. DRJ participated in study coordination, statistical analysis and helped to draft the manuscript. MD conceived of the study and obtained funding, participated in its design and coordination and helped to draft the manuscript. All authors read and approved the final manuscript.

## Pre-publication history

The pre-publication history for this paper can be accessed here:

http://www.biomedcentral.com/1471-2288/10/2/prepub
